# MYC—an emerging player in mitochondrial diseases

**DOI:** 10.3389/fcell.2023.1257651

**Published:** 2023-09-04

**Authors:** Janne Purhonen, Juha Klefström, Jukka Kallijärvi

**Affiliations:** ^1^ Folkhälsan Research Center, Helsinki, Finland; ^2^ Stem Cells and Metabolism Research Program, Faculty of Medicine, University of Helsinki, Helsinki, Finland; ^3^ Finnish Cancer Institute, FICAN South Helsinki University Hospital, Helsinki, Finland; ^4^ Translational Cancer Medicine, Medical Faculty, University of Helsinki, Helsinki, Finland; ^5^ Department of Cell and Tissue Biology, University of California, San Francisco, San Francisco, CA, United States

**Keywords:** electron transport chain, oxidative phosphorylation, respiratory complex III, mitochondrial integrated stress response, Warburg effect, cellular senescence

## Abstract

The mitochondrion is a major hub of cellular metabolism and involved directly or indirectly in almost all biological processes of the cell. In mitochondrial diseases, compromised respiratory electron transfer and oxidative phosphorylation (OXPHOS) lead to compensatory rewiring of metabolism with resemblance to the Warburg-like metabolic state of cancer cells. The transcription factor MYC (or c-MYC) is a major regulator of metabolic rewiring in cancer, stimulating glycolysis, nucleotide biosynthesis, and glutamine utilization, which are known or predicted to be affected also in mitochondrial diseases. Albeit not widely acknowledged thus far, several cell and mouse models of mitochondrial disease show upregulation of MYC and/or its typical transcriptional signatures. Moreover, gene expression and metabolite-level changes associated with mitochondrial integrated stress response (mt-ISR) show remarkable overlap with those of MYC overexpression. In addition to being a metabolic regulator, MYC promotes cellular proliferation and modifies the cell cycle kinetics and, especially at high expression levels, promotes replication stress and genomic instability, and sensitizes cells to apoptosis. Because cell proliferation requires energy and doubling of the cellular biomass, replicating cells should be particularly sensitive to defective OXPHOS. On the other hand, OXPHOS-defective replicating cells are predicted to be especially vulnerable to high levels of MYC as it facilitates evasion of metabolic checkpoints and accelerates cell cycle progression. Indeed, a few recent studies demonstrate cell cycle defects and nuclear DNA damage in OXPHOS deficiency. Here, we give an overview of key mitochondria-dependent metabolic pathways known to be regulated by MYC, review the current literature on MYC expression in mitochondrial diseases, and speculate how its upregulation may be triggered by OXPHOS deficiency and what implications this has for the pathogenesis of these diseases.

## 1 Introduction

**FIGURE 1:**
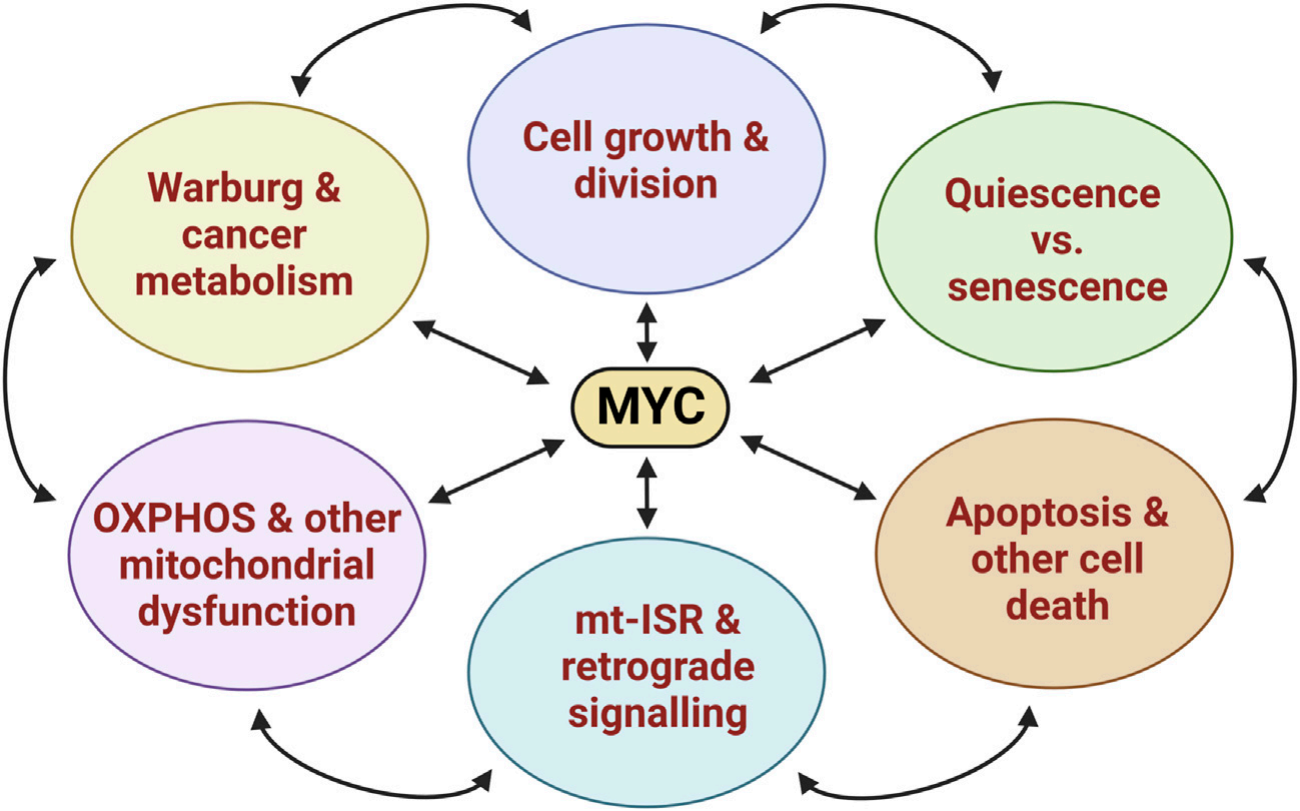
The cartoon illustrates some central physiological processes in mammalian cells and tissues to which MYC-regulated transcriptional programs contribute as driver, modulator, or adaptor. The abbreviation mt-ISR stands for mitochondrial integrated stress response. See sections 3-5 in the main text for elaboration.

MYC (c-MYC, avian MYeloCytomatosis viral oncogene homolog) is a Basic-Helix-Loop-Helix-Leucine Zipper (bHLHZip)-family transcription factor that regulates a broad range of cellular functions including metabolism, growth, proliferation, differentiation, and survival (Figure 1) (Hartl, 2016). It is a major driver of cancer, being frequently overexpressed but rarely mutated. Transgenic overexpression of MYC in mice leads to increased proliferation and tumor development in multiple tissues. Conversely, inhibition or removal of MYC consistently causes growth arrest of cancer cells both in culture and *in vivo* (Dave et al., 2017). In mammals, the MYC family also includes MYCN (N-MYC) and MYCL (L-MYC) proteins, which are highly homologous to MYC but expressed spatially differently. MYC is expressed widely but at a very low level in non-proliferating (quiescent) or postmitotic cells, whereas it is typically much more abundant in proliferating cells. Semsei et al. studied the tissue expression of *Myc* throughout mouse development and life span (Semsei et al., 1989). Its expression was highest in prenatal and newborn tissues and then decreased, reaching its lowest levels at about 6 months. The spleen and liver consistently showed the highest *Myc* expression at all ages. Interestingly, *Myc* expression did not continue to decline upon further ageing but instead progressively increased in the brain, liver, skin, and small intestine.

In contrast to the broad but low-level expression of MYC, MYCN expression mainly limits to neuronal and reproductive tissues, whereas MYCL expression restricts to the gastrointestinal tract, including pancreas and dendritic cells (Das et al., 2023). MYC and MYCN germline knockouts are embryonic lethal, whereas mice lacking MYCL do not have an overt phenotype. All three MYC family members heterodimerize with MAX (MYC-Associated factor X) to exert their transcriptional functions. MAX can homodimerize or heterodimerize with other proteins than MYC family, and these dimers compete for a common DNA sequence element called the E box, providing a complex transcriptional regulation system. After DNA binding, the MYC-MAX dimers can recruit further transcriptional cofactors and chromatin modiflers that license RNA polymerase II activation and transcription. The MYC-driven transcriptional programs are complex and almost genome wide because MYC acts as a global transcriptional amplifier that binds and increases expression at active promoters (Nie et al., 2020). Thus, MYC disproportionally upregulates highly expressed genes. Notwithstanding that its modes of conducting transcriptional activation are still somewhat obscure despite intensive research, MYC has been estimated to control the expression of at least 15% of all genes in humans, some of the most prominent categories being genes involved in cell cycle progression, metabolism, ribosomal biogenesis, and translation (Hartl, 2016).

The MYC-MAX dimerization domain function is conserved from human to zebrafish (Schreiber-Agus et al., 1993) and fruit fly (Schreiber-Agus et al., 1997). The discovery of MYC and MAX homologs in the unicellular organisms choanoflagellates revealed that MYC evolved even before the metazoa (animals) (Young et al., 2011). In *Hydra* (polyp), *Myc* mRNA is highly expressed in stem cells and other rapidly proliferating cell types, whereas in terminally differentiated cells, its expression is not detectable, suggesting an overall conserved role in cell proliferation (Hartl et al., 2010). The fruit fly (*Drosophila melanogaster*) MYC homolog (dMyc, diminutive) (Schreiber-Agus et al., 1997) has been studied quite extensively in the context of normal physiology. Loss of dMyc function impedes growth and reduces cell size, whereas dMyc overexpression boosts growth and cell size and promotes G_1_/S progression but not G_2_/M progression or cell division (Johnston et al., 1999). It also increases genomic rearrangements, typical of erroneous DNA double-strand break repair, and shortens lifespan. Conversely, *dMyc* haploinsufficiency decreases mutation load and extends lifespan (Greer et al., 2013), similarly to in mice (Hofmann et al., 2015), as we shall see in the next section.

## 2 Insight into normal MYC function and regulation from genetic models

Despite the extensive knowledge about the roles of MYC in carcinogenesis and cultured cancer cells accumulated during the past 4 decades, much less is still known about its roles in normal development and tissue homeostasis (Figure 1). Greatly aiding studies on the latter, the homologous recombination-based gene knockout (KO) technology allowed the development of several invaluable genetic models to assess MYC function during ontogenesis (Table 1). In the early 1990s, it turned out that homozygous *Myc* knockout in mice is lethal by embryonic day 10.5 due to developmental defects in the placenta, hematopoietic system, and vasculature (Davis et al., 1993). Embryonic fibroblasts (MEFs) isolated from E9.5 KO embryos are viable but flattened and do not proliferate (Trumpp et al., 2001). A highly useful early *Myc* KO *in vitro* model was developed via homologous recombination in rat fibroblasts (Mateyak et al., 1997). These cells are highly abnormal and divide 2 to 3 times more slowly than controls, are very flattened, and show dramatically decreased or delayed cyclin D, E and A expression during the cell cycle. Their mitochondria are less abundant, much smaller in size, and show disrupted cristae patterns, indicating that MYC plays a role in the maintenance of mitochondria. Furthermore, Myc KO fibroblasts show decreased glycolysis, mitochondrial membrane potential and respiration, and low levels of oxidative phosphorylation (OXPHOS) machinery enzymes, likely explaining their 3-fold decreased ATP level.

**TABLE 1:** Models with genetic MYC manipulation and their effect on mitochondria.

**Model/allele**	**Effect on MYC**	**Phenotype**	**Mitochondria-related changes**	**References**
***Myc* KO in rat normal fibroblasts**	KO	Decreased growth rate, doubling time 4-5 days (WT 18-24 h)	Decreased number, size, membrane potential, OXPHOS, ATP level, CI, ATP synthase, SCs, fusion	Mateyak et al. (1997), Graves et al. (2012)
**Heterozygous exon 2&3 excision (*Myc*** ^***+/−***^ **) *in vivo***	mRNA, protein 50% of WT	Increased longevity and health span, unaltered cancer incidence	Decreased fatty acid and cholesterol synthesis	Hofmann et al. (2015)
**Excised exons 2&3 with *Alb-Cre in vivo***	>90% KO in hepatocytes	No overt phenotype	Mild respiration defect in liver mitochondria. Increased FAO.	Edmunds et al. (2016)
**Excised exons 2&3 with *ROSA26-CreER***	Whole body 75%-95% loss of mRNA expression	Signs of accelerated aging, fatty liver disease, decreased incidence of tumours	Increased whole-body FAO, decreased CI activity, increased ROS in MEFs	Wang et al. (2023)
**(tamoxifen) *in vivo***				
**Excised exons 2&3 with *ROSA26-CreER* (tamoxifen) in MEFs**	>95% loss of MYC protein	Poor proliferation, G_o_/G_1_ arrest, flattened senescent morphology, DNA damage response	Increased mitochondrial mass, ROS production	Wang et al. (2022)

CI, complex I; SCs, supercomplexes; FAO, fatty acid oxidation; ROS, reactive oxygen species; MEFs, mouse embryonic fibroblasts.

In contrast to complete or near-complete *Myc* loss-of-function, *Myc* haploinsufficiency (*Myc* ^*+/−*^) brings no adverse effects but about 20% decrease in adult body mass and increased lifespan in mice (20% in females, 10% in males) (Hofmann et al., 2015). This increased longevity is accompanied by a lower incidence of age-associated pathologies such as osteoporosis, cardiac fibrosis, and immunosenescence. Compared to wild-type mice, the *Myc* ^*+/−*^ mice are also more active and have a higher metabolic rate. Upregulation of ribosome biogenesis is a canonical MYC-driven process, and indeed the *Myc* ^*+/−*^ mice have slightly reduced ribosomal RNA content and protein translation. The mice also have reduced serum IGF-1, increased AMPK activity, and decreased AKT and mTOR activities, altogether indicating a marked suppression of anabolic metabolism.

Despite the importance of MYC for embryonic and cancer growth, several studies have shown that MYC is largely dispensable for normal tissue growth and homeostasis in juvenile and adult stages. In adult human and mouse tissues, MYC expression is highest in rapidly proliferating compartments like the intestinal crypts and skin (Dave et al., 2017). However, conditional deletion of *Myc* in these compartments in mice does not result in a noticeable proliferation defect. For example, quite unexpectedly, MYC is dispensable for postnatal liver growth and regeneration (Edmunds et al., 2016). Nevertheless, transient induction of MYC occurs upon induced liver regeneration in various rodent liver injury models such as partial hepatectomy and hepatotoxic drug treatment (Prochownik, 2022). However, MYC’s role in such experimental models of liver regeneration is less clear. In some models, the pace of liver mass restoration was unaffected by conditional loss of MYC in hepatocytes (Li et al., 2006; Sanders et al., 2012), while others claim that regeneration is compromised (Baena et al., 2005). Utilizing fumaroylacetoacetate hydrolase (FAH) mutant mice, a genetic liver disease model mimicking human type I hereditary tyrosinemia, subjected to transplantation of WT and *Myc* ^*−/−*^ hepatocytes, Edmunds et al. showed convincingly that MYC is dispensable for liver regeneration in this model (Edmunds et al., 2016).

To assess the regulation of *Myc* transcription in normal tissues and upon tumorigenesis, Dave et al. generated a series of alleles with large deletions in the *Myc* 5’ regulatory sequences (Dave et al., 2017). The mice carrying the largest (>500 kb) of these (*Myc* ^*△2–540*^ **)** show a 50%–80% decrease in basal *Myc* expression but are homozygous viable and fertile, with no overt phenotype and only a slight decrease in the number of B lymphocytes. These mice fail to induce MYC overexpression during early tumorigenesis, and cultured fibroblasts from these mice grow slowly and are unable to upregulate MYC in response to serum stimulation. In a recent paper, Wang et al. revisit the question of MYC functions in postnatal development and tissue homeostasis by generating a mouse line with tamoxifen-inducible Cre-mediated loss of *Myc* (Wang et al., 2023). Their system allowed near-complete (75%-95%) elimination of *Myc* expression with a 5-day tamoxifen regimen started at 4 weeks of age. Somewhat surprisingly, the resulting mice with extreme but not complete loss of MYC display some premature aging features, like alopecia and graying of the hair as early as 3-4 months of age, increased adiposity, and hepatic steatosis. However, the mice live significantly (median 4.6 months) longer than wild-type controls, probably due to a 4-5-fold lower cancer incidence. Transcriptional profiling of the liver, white adipose tissue and skeletal muscle showed changes related to mitochondrial and ribosomal function, cellular senescence, DNA damage recognition and repair, and mRNA splicing upon loss of MYC (Wang et al., 2023). The results from both hypomorphic mouse models indicate that MYC is an important metabolic regulator but not critical for the normal physiology of adult mice.

## 3 Regulation of normal mitochondrial homeostasis and metabolism by MYC

As the main cellular site of energy metabolism and biosynthesis, mitochondria are highly dynamic and respond to the selection of available nutrients and requirements for biomass production to ensure sufficient resources for cell proliferation, tissue growth, homeostasis, and mechanical work. MYC is a major driver of cellular proliferation and growth, so it is obvious that the transcriptional programs driven by it must be tightly connected to mitochondrial homeostasis, energy metabolism and biosynthesis (O’Connell et al., 2003; Morrish and Hockenbery, 2014; Goetzman and Prochownik, 2018). To understand why and how MYC might be needed in response to mitochondrial dysfunction, such as in mitochondrial diseases, we will take a brief look at what is known about how MYC regulates some key mitochondria-related functions. It is worth bearing in mind, however, that most of these findings were made in cancer studies, or at least using cancer cell lines as a model.

### 3.1 Mitochondrial dynamics and biogenesis

Mitochondrial biogenesis generates new mitochondria to maintain or increase mitochondrial number and mass in a cell (Morrish and Hockenbery, 2014). It is a massive undertaking requiring the transcription, translation, mitochondrial import, and assembly of over 1,000 nuclear genome-encoded proteins and 13 proteins encoded by the mitochondrial genome (mtDNA). Numerous studies employing MYC-overexpressing or MYC-deleted cell systems have shown that nearly half of the nuclear genes encoding mitochondrial proteins can be transcriptionally upregulated by MYC (Li et al., 2005; Kim et al., 2008; Graves et al., 2012; Morrish and Hockenbery, 2014). In addition to transcript level analyses, Li. et al. demonstrated, utilizing a human lymphoblastoid cell line carrying tetracycline-repressible ectopic *MYC* and estradiol-inducible endogenous *MYC,* that MYC overexpression increases mitochondrial mass and lack of MYC has the opposite effect (Li et al., 2005).

Mitochondria cannot be built from scratch, but “new” mitochondria are always generated from existing ones by means of fission and fusion of the organelle coupled to mtDNA replication, transcription and translation. A generally accepted purpose of fusion is to mitigate stress and maximize function by mixing the contents of damaged and healthy mitochondria. In contrast, fission is a means to create new mitochondria and a quality control mechanism to remove damaged mitochondria via mitophagy (Goetzman and Prochownik, 2018). Expression of many mitochondrial proteins controlling fission and fusion, such as the mitofusins Mfn1 and Mfn2, is low in *Myc* ^*−/−*^ fibroblasts compared to the cells rescued by *Myc* transfection (Graves et al., 2012). MYC-deficient cells also have twofold lower rates of mitochondrial fusion compared to MYC-expressing cells, suggesting that the normally proliferating MYC-expressing cells were under pressure to ensure mitochondrial quality for sufficient energy and biosynthetic precursor production (Wang et al., 2022).

One of the first identified transcriptional targets of MYC encoding a mitochondrial protein was SURF-1, an assembly factor for respiratory complex IV (cytochrome c oxidase, CIV) (Vernon and Gaston, 2000). Direct MYC target genes also involve several other respiratory complex assembly factors, the TIM/TOM (Translocase of the Inner/Outer Membrane) proteins, and practically all mitochondrial ribosomal proteins (Morrish and Hockenbery, 2014). Most of the major transcription factors that drive mitochondrial biogenesis in response to metabolic cues are transcriptional targets of MYC, at least in some systems (Seitz et al., 2011). The best-characterized ones are TFAM (Transcription factor a, mitochondrial, a key regulator of mtDNA transcription and replication), PPARGC1A (Peroxisome proliferator-activated receptor γ coactivator 1-α, also known as PGC-1α), ESRRA/B/G (Estrogen-related receptors α/β/γ, also known as ERRα/β/γ), PPARA/D/G (Peroxisome proliferator-activated receptors α/δ/γ), NRF1 (Nuclear respiratory factor 1), GABPB1 (also called Nuclear respiratory factor 2), and PPRC1 (PPARG-related coactivator 1). Because of several reviews on the roles of these TFs in mitochondrial biogenesis (Dinkova-Kostova and Abramov, 2015; Gureev et al., 2019; Popov, 2020; Vernier and Giguère, 2021), it suffices to say here that their interplay with MYC in the context of OXPHOS dysfunction is an understudied but exciting topic.

### 3.2 Energy metabolism and biosynthesis

Cellular metabolism can be divided into catabolic (degradative) and anabolic (biosynthetic) branches that intertwine widely. Catabolism is the collective term for breaking complex molecules into simpler ones with concomitant release of energy from chemical bonds to drive cellular functions and to produce biosynthetic precursors. Uncovering the central role of MYC as a regulator of both catabolism and anabolism started with studies on cancer cells in the mid-1980s. To begin with, MYC plays a crucial role in the regulation of glycolysis by upregulating glucose transporters and nearly all the glycolytic enzymes and by regulating pyruvate kinase splicing (Haikala et al., 2017; Goetzman and Prochownik, 2018; Dong et al., 2020). Indeed, the basal rate of glycolysis in the aforementioned *Myc* ^*−/−*^ fibroblasts is about 50% of that of parental wild-type cells (Graves et al., 2012). An example of a canonical anabolic process driven by MYC is the upregulation of ribosome biogenesis and protein synthesis (van Riggelen et al., 2010).

Proliferating cells require a continuous supply of amino acids, nucleotides, and lipids as building blocks of cell mass. Apart from tumor cells this goes for embryonic and adult stem cells as well as differentiated cells, such as hepatocytes, that can enter the cell cycle for tissue regeneration. All these cell types share a reliance on glucose and glutamine to support their anabolism (Haikala et al., 2017; Goetzman and Prochownik, 2018). Glutamine is the most abundant amino acid in human blood, and proliferating cells use it for energy production and as a carbon and nitrogen source for biosynthesis. MYC promotes glutamine metabolism directly and indirectly via diverse mechanisms, for example, through upregulation of glutamine synthesis, uptake, transport, and consumption. A recent review thoroughly covers the genetic and biochemical mechanisms of regulation of glutamine uptake and metabolism by MYC (Tambay et al., 2021). As the first step of glutaminolysis, the hydrolase enzymes glutaminase 1 and 2 (GLS-1, GLS-2) convert glutamine to glutamate. MYC can upregulate GLS-1 via miR-23a/b-dependent posttranscriptional mechanisms (Gao et al., 2009) and also more directly via binding to the *GLS-1* transcription start site near the 5′UTR (Haikala et al., 2018). In the cytosol, glutamate serves as a substrate for the synthesis of several amino acids (serine, alanine, aspartate, and ornithine), whereas it mainly undergoes conversion to α-ketoglutarate in the mitochondria to fuel the Krebs cycle (Haikala et al., 2017).

In contrast to proliferating cells, many differentiated permanently postmitotic tissues prefer to utilize fatty acid oxidation (FAO) over glycolysis for energy production. Loss or inhibition of MYC leads to exaggerated reliance on FAO as an energy source in several cell and tissue models (Graves et al., 2012; Zirath et al., 2013; Edmunds et al., 2014; 2016). Finally, one canonical target of MYC is nucleotide metabolism, and MYC directly binds the regulatory regions of many genes encoding enzymes involved in purine and pyrimidine nucleotide biosynthesis (Liu et al., 2008). Moreover, MYC upregulates pathways such as *de novo* serine synthesis and one-carbon metabolism that support nucleotide biosynthesis (Sun et al., 2015).

### 3.3 The Warburg effect

**FIGURE 2:**
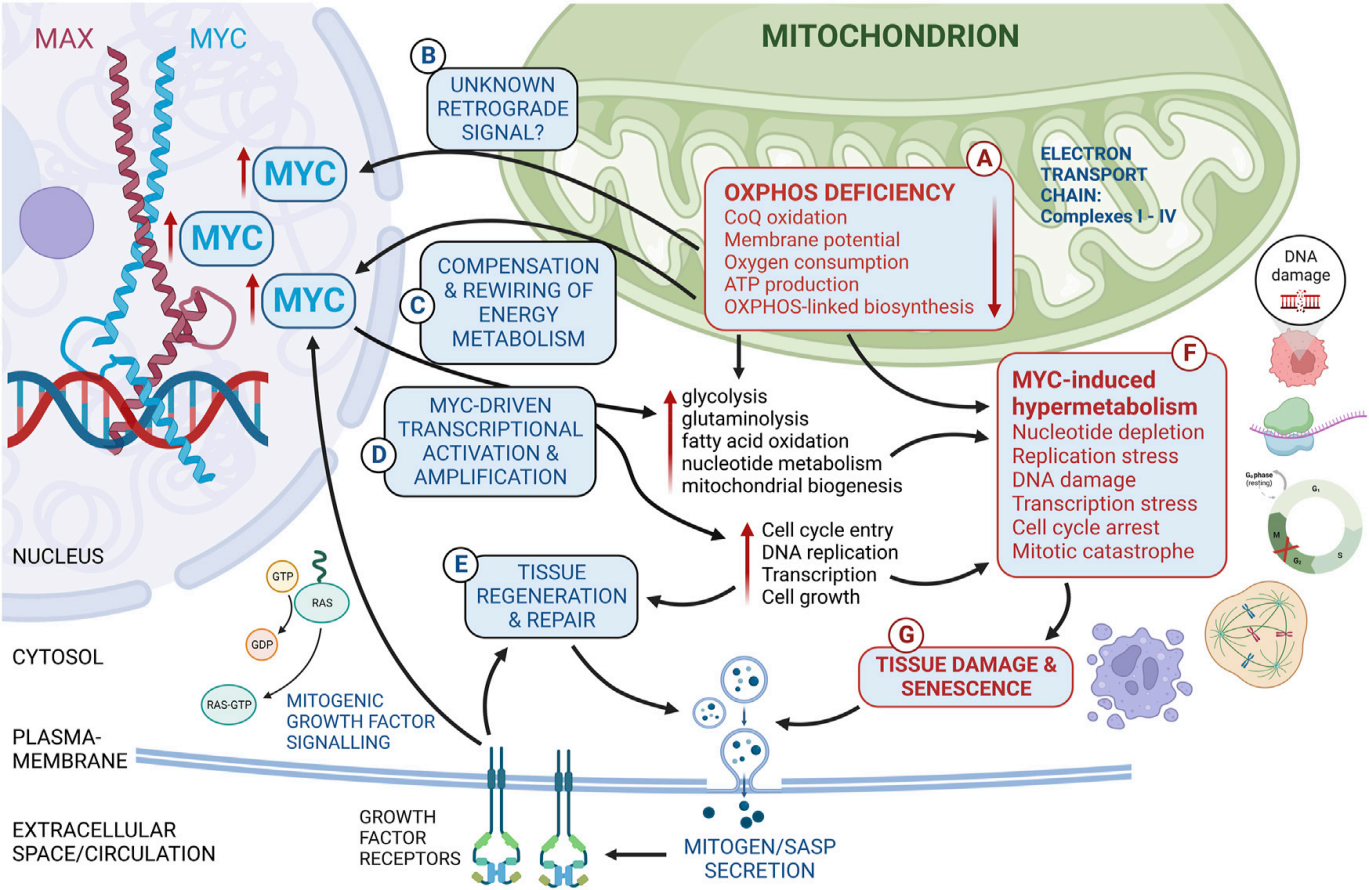
Cartoon showing connections between OXPHOS deficiency, MYC, cellular metabolism, and cell fates. Mitochondrial diseases are genetic diseases, in which a mutation in either the nuclear or mitochondrial genome compromises directly or indirectly mitochondrial ATP production by the oxidative phosphorylation (OXPHOS) machinery **(A)**. OXPHOS deficiency triggers a retrograde (mitochondrion-to-nucleus) signal **(B, C)** to adjust gene expression to restore homeostasis (anterograde signals, **(C, D)**) and to allow cell survival and tissue regeneration and repair **(E)**. The nature of the retrograde signal **(B)** triggering MYC expression in OXPHOS deficiency remains uncertain. Beneficial or neutral compensatory changes induced by OXPHOS deficiency are shown in text boxes with blue font **(C–E)**. Known or hypothetical adverse consequences of MYC induction concomitantly with OXPHOS deficiency are listed in text boxes with red font **(F, G)**. These include hypermetabolism (F, increased demand for energy and biosynthesis), potentially leading to nucleotide depletion, cell cycle arrest, genomic instability, and senescence **(G)** with senescence-associated secretory phenotype (SASP).

In mitochondrial diseases, cells unavoidably rewire their metabolism to compensate for the compromised OXPHOS (Figure 2). In cancer cells, glycolysis coupled to lactate production is often favored over OXPHOS even in the presence of sufficient oxygen, an effect first observed by Otto Warburg in the 1920s. Such “fermenting glycolysis” was initially suggested by Warburg to be caused by defective mitochondria (Warburg, O. et al., 1924; Warburg, 1956). His contemporary Herbert Crabtree suggested an opposite explanation: that cancer cells downregulate OXPHOS in response to increased glycolysis (Crabtree, 1929). The Warburg effect is now known to occur in both rapidly proliferating normal cells and cancer cells without any impairment in OXPHOS (Senyilmaz and Teleman, 2015; Goetzman and Prochownik, 2018; Vaupel and Multhoff, 2021). Instead, it is essentially a metabolic reprogramming resulting from characteristic normal and/or malignant proliferation-associated transcriptional and signaling alterations, such as hypoxia-inducible factor-1 (HIF-1) stabilization, oncogene activation (MYC, Ras), loss of function of tumour suppressors (P53, PTEN), activated (PI3K-Akt-mTORC1, RAS-RAF-MEK-ERK, Jak-Stat3), or deactivated (LKB1-AMPK) energy signaling pathways (Senyilmaz and Teleman, 2015; Goetzman and Prochownik, 2018; Vaupel and Multhoff, 2021). At the molecular level, some of the key features of the Warburg effect are accelerated glycolytic flux, diversion of glycolytic intermediates to the biosynthesis of nucleotides, non-essential amino acids, lipids, and hexosamines, inhibition of pyruvate entry into mitochondria, increased production of lactate from pyruvate, secretion of lactate through lactate–proton symporters, and increased carbonic anhydrase activity to hydrate CO_2_ from oxidative metabolism into H^+^ and bicarbonate. Excessive lactate-proton export results in extracellular acidification, which may drive further malignant cancer progression (Cassim et al., 2020; Vaupel and Multhoff, 2021). In the case of mitochondrial disease, decreased tricarboxylic (Kreb’s) cycle activity may decrease pyruvate oxidation, force its reduction to lactate, and result in the relatively common manifestation lactic acidemia (Hikmat et al., 2021). Even though mutations compromising OXPHOS do not underlie the Warburg effect in cancer cells, mutations causing mitochondrial diseases lead to metabolic changes that are overlapping with it. Whether this might have implications for the pathogenesis of these diseases, particularly those affecting proliferating or regeneration-competent tissues, remains to be studied. In the next section, we shall turn to what is currently known about MYC in experimental OXPHOS dysfunction and mitochondrial disease models.

## 4 Upregulation of MYC in OXPHOS dysfunction

Even though not widely acknowledged, mRNA expression data from several cell and mouse models of mitochondrial dysfunction show upregulation of MYC (summarized in Table 2). In the earliest of these, Miceli and Jawlinski studied the response in nuclear gene expression to loss of mtDNA (ρ0 or “rho zero” cells) in two human cell lines (T143B osteosarcoma and ARPE19 retinal pigment epithelium) and fibroblasts from an individual with Kearns-Sayre syndrome (KSS, mitochondrial myopathy due to inherited mtDNA deletions). *MYC* was among the genes commonly induced due to the loss of mtDNA in all 3 cell models. RNA interference experiments in the ARPE19 cells suggested that the induction of *MYC* was related to the upregulation of glycolysis (Miceli and Jazwinski, 2005). Cortopassi et al. performed a microarray profiling of 22 different cell lines (including lymphoblasts, fibroblasts, myoblasts, muscle, and osteosarcoma cybrids) representing five mitochondrial diseases: Leber’s Hereditary Optic Neuropathy (LHON), Friedreich’s ataxia (FRDA), Mitochondrial encephalomyopathy, lactic acidosis and stroke-like episodes (MELAS), KSS, and Neurogenic ataxia and retinitis pigmentosa (NARP). The authors reported a median 1.5-fold upregulation of *MYC* in 5/22 groups of cells. They also reported upregulation of cell cycle progression- and ribosomal biogenesis-associated genes and speculated that MYC may be driving these in mitochondrial dysfunction caused by ischemia or mutations (Cortopassi et al., 2006). Ten years on and with major technical advances in high-throughput technologies (“omics”), Kühl et al. moved to *in vivo* level. They performed an impressive transcriptomics and proteomics study of the heart tissue from five conditional knockout mouse strains that develop OXPHOS deficiency and cardiomyopathy due to impaired mtDNA gene expression (*Twnk*, *Tfam*, *Polrmt*, *Lrpprc* and *Mterf4* cKO alleles). The survival of these mice varied from 6 to 21 weeks. In end-stage hearts of all five cardiospecific knockouts, *c-Myc* was induced 4-12-fold. The authors also reported the upregulation of several known target gene sets of MYC, the most highly upregulated of them being related to the one-carbon metabolism. Kühl et al. did not study the role of MYC beyond mRNA expression but devoted a chapter to discussing the implications of this finding. In brief, they propose that MYC contributes to the remodeling of metabolism under severe mitochondrial dysfunction (Kühl et al., 2017).

**TABLE 2:** Current evidence for MYC upregulation in models of mitochondrial disease or dysfunction.

**Model/modified gene**	**Tissue/cell line**	**OXPHOS activity**	**MYC expression/targets**	**References**
**Chemical depletion of mtDNA (ρ** ^**0**^ **cells)**	T143B and ARPE19 cell lines, KSS fibroblasts	n.d. but presumed total loss	*MYC* mRNA up ∼1.5-fold (qPCR); targets n.d	Miceli and Jazwinski (2005)
***FRDA, KSS, LHON, MERRF, NARP* mutations**	primary patient fibroblasts	n.d	*MYC* mRNA up 1.5-fold (qPCR); targets n.d	Cortopassi et al. (2006)
**OXPHOS inhibitor treatment**	U2OS cells	n.d. but presumed loss	MYC protein up (WB)	Gleyzer and Scarpulla (2016)
***Twnk, Tfam, Polrmt, Lrpprc* and *Mterf4* cardiospecific KO mice (*Ckmm-Cre*)**	heart	n.d	*Myc* mRNA up 4-12-fold (transcriptomics); targets n.d	Kühl et al. (2017)
***Uqcrfs1* conditional KO (*Vav-iCre*)**	fetal hematopoietic stem cells	Decreased by ∼80% in fetal liver Lin^−^ cells	MYC targets the most highly upregulated gene signature	Ansó et al. (2017)
**Tamoxifen-induced Cre-mediated loss of *Uqcrq***	primary lung endothelial cells	n.d	MYC targets the most highly upregulated gene set	Diebold et al. (2019)
***Bcs1l*** ^***p.S78G***^ **and *Bcs1l*** ^***p.S78G***^ ***;mt-Cyb*** ^***p.D254N***^ **mice**	liver, kidney, heart, skeletal muscle (P21-150)	CIII activity 10%-50% of WT	MYC mRNA and protein up 2-40-fold (transcriptomics, qPCR, WB); cell cycle, nucleotide and one carbon metabolism up	Purhonen et al., 2017 (2023)
***Clpp* and *Fgf21* DKO mice**	heart	Mild decrease	*Myc* mRNA up 2.5-fold (transcriptomics)	Croon et al. (2022)
**Mitochondrial ribosomal protein S5 *(Mrps5)* cardiospecific KO**	heart	n.d	∼30% increase in MYC protein	Gao et al. (2023)

Chandel’s group studied the role of OXPHOS in hematopoietic stem cell maintenance in mice by deleting the respiratory complex III (CIII) subunit UQCRFS1 from mid-gestation (Ansó et al., 2017). Transcriptome analysis of isolated fetal liver hematopoietic stem cells revealed that MYC targets were the most significantly upregulated pathway upon the loss of CIII function. However, the authors did not discuss the implications of this finding. MYC came up also in another study by this group, investigating the role of endothelial cell energy metabolism in angiogenesis. To this end, they generated a mouse model with endothelial cell-specific tamoxifen-inducible loss of the CIII subunit UQCRQ. Induction of Cre expression immediately after birth resulted in lethality between 2 and 4 weeks of age due to impaired angiogenesis. Transcriptomics from CIII-deficient primary lung endothelial cells isolated from these mice revealed significant upregulation in pathways associated with anabolism and cellular proliferation, including MYC target genes (Diebold et al., 2019). Additional circumstantial evidence for the involvement of MYC in mitochondrial disease pathogenesis came from a study assessing the regulation of the mitochondrial integrated stress response by the mitokine FGF21 in mitochondrial cardiomyopathy (Croon et al., 2022). In mitochondrial matrix protease *Clpp* KO heart, showing mild OXPHOS deficiency, *Myc* mRNA was ∼2.5-fold upregulated and this was blunted by FGF21 loss.

We first noted several years ago that in transcriptomics data from 6-week-old CIII-deficient *Bcs1l* ^*p.S78G*^ knock-in mice, *Myc* expression was highly upregulated (12-fold) and that it was the top predicted transcriptional regulator explaining the overall gene expression changes induced by CIII deficiency in the liver (Purhonen et al., 2017). These mice carry the GRACILE syndrome patient mutation, causing one of the most severe known OXPHOS deficiency phenotypes with fetal onset (Fellman et al., 1998; Hikmat et al., 2021). In five months-old *Bcs1l* ^*p.S78G*^ mice, the *Myc* induction wanes, but the mRNA is still significantly upregulated in the liver (5.4-fold), kidney (4.1-fold), and heart (2.3-fold), the three tissues studied. Notably, the heart is presymptomatic at this age, dilating cardiomyopathy developing by postnatal day 200 (P200) (Rajendran et al., 2019). We recently (Purhonen et al., 2023) extended the studies on the MYC upregulation into juvenile (postanal day 21–35) *Bcs1l* ^*p.S78G*^ mice and found a staggering level of MYC induction, 30-40-fold, in the P30 liver (symptomatic) at both mRNA and protein level. In the P30 kidney (symptomatic), *Myc* mRNA was upregulated about 10-fold. In the skeletal muscle, which has very low CIII activity (∼25% of wild-type) but no clear myopathy (Purhonen et al., 2020), *Myc* mRNA was upregulated more modestly, about 2-fold (Purhonen et al., 2023). Interestingly, MYC was induced presymptomatically, immediately after weaning (P18-P25) in the liver and possibly also in the other tissues. MYC is known to be transiently induced in liver regeneration, but the level of induction in the CIII-deficient mice was at least an order of magnitude higher than in typical liver injury models, such as 2/3 hepatectomy and bile duct ligation (Sekine et al., 2007; Zhang et al., 2019), despite that their liver disease is relatively mild when the MYC upregulation first appears. This suggests a mechanism for the MYC induction by CIII deficiency that is not solely related to the tissue regeneration need.

## 5 Is MYC a component of the mitochondrial retrograde signal and/or the integrated stress response (mt-ISR)?

MYC is seldom mutated in cancer but rather upregulated via transcriptional induction due to chromosomal translocation or gene copy number amplification. Alternatively, MYC can be activated by excessive growth factor signaling due hyperactivating mutations in or amplification of growth factor receptors (such as the epidermal growth factor receptor, EGFR) or signaling proteins such as Ras (Hartl, 2016). Again, much less is known about the induction mechanism of MYC in non-cancerous diseases but, presumably, the above-mentioned mitogenic signaling mechanisms, hijacked by cancer cells, are at play. How and why would mitochondrial dysfunction lead to MYC induction? In the liver of the juvenile *Bcs1l* ^*p.S78G*^ mice, we see 50- to 600-fold upregulation of the EGFR ligand amphiregulin (AREG) (Purhonen et al., 2023), a recently identified mitokine (Hino et al., 2022). A simple explanation would be that upregulation of EGFR ligands due to tissue growth and regeneration pressure drives Ras-MAPK signaling and the MYC induction in the CIII-deficient tissues (Figure 2).

Aside from the canonical growth factor signaling paradigm, there are some other possibilities for how mitochondria could communicate more directly with the nucleus to regulate MYC expression (Figure 2). Retrograde signaling refers to the process where a signal travels backwards from an organelle to the nucleus (Bilen et al., 2022). For example, signals from the mitochondria are relayed to the nucleus via small molecules and/or proteins and/or protein modifications to regulate nuclear gene expression. The mitochondrion-nucleus retrograde signaling has been thoroughly characterized in the yeast (*Saccharomyces cerevisae*). In this organism, Retrograde regulation protein 1 (Rtg1), the key conveyor of mitochondrial stress signals to the nucleus, has been suggested to be a MYC homolog (Jazwinski and Kriete, 2012). The yeast Rtg1 and Rtg3 proteins are bHLH/zip transcription factors that heterodimerize analogously to MYC and MAX (Jia et al., 1997b). Although their overall sequence similarity is low, structural modeling revealed conservation of key bHLH/zip residues between Rtg1 and MYC and Rtg3 and MAX (Srinivasan et al., 2010). In support of involvement of MYC in mammalian mitochondrial retrograde signaling, Gleyzer and Scrapulla found concurrent upregulation of the mitochondrial biogenesis-related transcription factor PGC-1-related coactivator (PRC) and MYC upon loss of OXPHOS, mainly induced by the protonophore CCCP in a human osteocarcinoma cell line U2OS (Gleyzer and Scarpulla, 2011; Gleyzer and Scarpulla, 2013; Gleyzer and Scarpulla, 2016). They also found that MYC induction is largely required for PRC stabilization and accumulation in response to mitochondrial stress (Gleyzer and Scarpulla, 2016). Furthermore, they showed that AKT phosphorylation-dependent steps are involved. Other key upstream players in MYC upregulation and universality of the findings of Gleyzer and Scrapulla in other cell lines and *in vivo* remain, however, yet to be clarified.

Various mitochondrial insults trigger a common transcriptional program called mitochondrial integrated stress response (mt-ISR) (Costa-Mattioli and Walter, 2020; Mick et al., 2020; Bilen et al., 2022). It has a resemblance to the more general integrated stress response (ISR), which is launched, for example, by amino acid starvation or ER stress. A convergent event in all integrated stress responses is the phosphorylation of Ser51 of the alpha subunit of eukaryotic initiation factor 2 (eIF2α), blocking 5′cap-dependent translation initiation. This results in suppressed global translation but increased translation of selected mRNAs that contain inhibitory upstream open reading frames, leading to translation of ISR mediators such as ATF4, ATF5, and CHOP (DDIT3). Noteworthily, *MYC* mRNA contains an internal ribosomal entry segment and an alternative in-frame start codon, enabling MYC translation under cellular stressors that suppress global protein synthesis (Subkhankulova et al., 2001). Four distinct serine/threonine kinases can perform the eIF2α phosphorylation: PKR (interferon-induced double-stranded RNA-dependent eIF2α kinase, induced by viral infection), PERK (PKR-like endoplasmic reticulum resident kinase, induced by endoplasmic reticulum stress), GCN2 (general control nonderepressible 2, induced by amino acid starvation), and HRI (heme-regulated inhibitor kinase, induced by heme deficiency but also by various other stressors) (Costa-Mattioli and Walter, 2020; Bilen et al., 2022). Of these, HRI undergoes activation in mitochondrial stress by OMA1-cleaved and retrotranslocated mitochondria-resident DELE1 protein (Fessler et al., 2020; Guo et al., 2020; Sekine et al., 2023). In addition, mitochondrial complex I (CI) inhibition can activate GCN2 due to asparagine depletion (Mick et al., 2020).

Mt-ISR drives an adaptive rewiring of metabolism to restore homeostasis. The most notable cellular processes that mt-ISR drives are *de novo* serine biosynthesis, one-carbon metabolism, and the transsulfuration pathway (Bao et al., 2016; Khan et al., 2017; Quirós et al., 2017). These processes are important, for example, for the synthesis of nucleotides, and phospholipids, and for the maintenance of redox balance. Not surprisingly, cancer cells exploit similar metabolic rewiring for survival and growth (Yang and Vousden, 2016). Most transcriptional programs driven by mt-ISR have been attributed to the transcription factor ATF4 (Quirós et al., 2017). However, very similar changes occur upon upregulation of MYC (Liu et al., 2008; Sun et al., 2015), which frequently accompanies mt-ISR (Table 2). Moreover, these two transcription factors share a high proportion of overlapping DNA-binding sites (Tameire et al., 2019). Observations from cancer cells that MYC-driven excessive anabolic metabolism can trigger ISR brings additional complexity into decoding ATF4- and MYC-driven transcriptional responses (Tameire et al., 2019).

Our data from the *Bcs1l* ^*p.S78G*^ mice with progressive loss of CIII function showed that, similar to cancer cell lines, MYC upregulation precedes eIF2-α phosphorylation (Purhonen et al., 2023). This observation suggests that MYC is not necessarily part of mt-ISR but potentially a component of a separate retrograde signaling and potential augmenter or even a trigger of ISR in these mice. In this study, we utilized transgenic *Ciona intestinalis* alternative oxidase (AOX) to interrogate the OXPHOS-dependent mechanisms. AOX is a mitochondrial enzyme from lower animals like yeasts, sea squirt (*C. intestinalis*) and plants and can transfer electrons directly from the coenzyme Q (CoQ) pool to oxygen when the CIII-CIV segment of the respiratory electron transfer is defective (Banerjee et al., 2021; Jacobs and Ballard, 2022). Surprisingly, we found that AOX robustly blunted the MYC-induction and mt-ISR—a highly paradoxical finding given that AOX did not improve any parameters directly linked to OXPHOS system such as ATP production and levels, mitochondrial membrane potential, or NADH/NAD^+^ ratio (Purhonen et al., 2023). Improved growth, prevention of liver and kidney pathology, and tripling of survival accompanied the suppressed MYC induction and mt-ISR in the AOX-expressing CIII-deficient mice. AOX also suppressed the MYC induction in the skeletal muscle, indicating that the mechanism is general also for postmitotic tissues.

## 6 Does MYC drive excessive anabolism and aberrant cell proliferation in mitochondrial diseases?

What are the consequences of MYC upregulation in tissues affected by mitochondrial disease? Some adaptive responses driven by MYC, such as mitochondrial biogenesis and enhanced glutaminolysis and glycolysis likely help cells to cope with defective OXPHOS. Nevertheless, in some sense, MYC upregulation is a paradoxical response to compromised energy metabolism due to the many energy-consuming processes it promotes. Elucidation of MYC’s beneficial adaptive and potentially pathological roles in mitochondrial diseases requires *in vivo* modulation of MYC. Very limited experiments to that end, however, exist. The most robust evidence for potential pathological role of MYC induction in mitochondrial disease comes from our studies on the CIII-deficient *Bcs1l* ^*p.S78G*^ mice*,* as reviewed above (Purhonen et al., 2023). A common feature of excessive MYC levels in both cancerous and normal cells is facilitated evasion of metabolic checkpoints, replication stress, DNA damage, and genomic instability (Felsher et al., 2000; Rohban and Campaner, 2015). Indeed, we showed that affected parenchymal cells in tissues that renew via cell cycle entry of differentiated cells, such as hepatocytes in the liver, induce a DNA damage response upon loss of CIII activity (Purhonen et al., 2023). As expected from the degree of MYC upregulation, severe nucleotide depletion did not suppress cell cycle entry or progression to the S-phase in the liver or kidney of *Bcs1l* ^*p.S78G*^ mice, suggesting MYC-driven illicit cell cycle progression. Similar to serum-starved fibroblasts forced to proliferate by MYC overexpression (Felsher et al., 2000), proliferating CIII-deficient hepatocytes of *Bcs1l* ^*p.S78G*^ mice showed cell cycle arrest at G2 phase and almost never reached mitosis. Those CIII-deficient hepatocytes that entered mitosis showed frequent aberrations such as multipolar mitotic spindles and anaphases, anaphase bridges, and lagging or dispersed chromatin, in other words cytological hallmarks of genomic instability. Suppression of MYC function with the dominant negative mutant fragment of MYC called Omomyc was sufficient to alleviate the DNA damage in CIII-deficient hepatocytes.

Inevitably, the replication issues in *Bcs1l* ^*p.S78G*^ mice led to widespread cellular senescence (Purhonen et al., 2023). Cellular senescence is often accompanied by senescence-associated secretory phenotype (SASP), which involves secretion of a variety cytokines, chemokines, growth factors, and proteases by the senescent cells (Xu et al., 2019). One possible driver or amplifier of the MYC upregulation in the *Bcs1l* ^*p.S78G*^ mice are the SASP-related EGFR ligands, the excessive secretion of which could lead to a circle of mitogenic stimulation (Figure 2). Mitochondrial stress as such can also induce the expression of the EGFR ligand amphiregulin (AREG) (Hino et al., 2022). Areg is one of most upregulated genes in the liver and kidney of the *Bcs1l* ^*p.S78G*^ mice (Rajendran et al., 2019; Purhonen et al., 2023).

Presumably, the blockade of mt-ISR and MYC induction by AOX prevented the CIII deficiency-induced changes in the expression of proliferation-associated genes, plunged proliferation markers to below WT level, and abolished DNA damage and cellular senescence. These findings indicate that limiting cell cycle entry can prevent tissue pathology caused by CIII deficiency (Purhonen et al., 2023). Intriguingly, we found that also low carbohydrate-high fat ketogenic diet, which we previously showed to have an unexpected beneficial effect on the liver disease in the *Bcs1l* ^*p.S78G*^ mice (Purhonen et al., 2017), dampened the MYC induction, limited the DNA damage, and moderated excessive hepatocyte proliferation (Purhonen et al., 2023). Further studies are, however, needed to elucidate therapeutic effects of MYC inhibition in this model. In the whole organism, the above-described pathological issues of cellular proliferation result in a premature aging (progeroid) disease similar to laminopathic and DNA repair-deficient juvenile progeroid syndromes. Interestingly, several other progeroid phenotypes due to mutations in genes encoding mitochondrial proteins have been reported relatively recently (Ehmke et al., 2017; Writzl et al., 2017; Elouej et al., 2020; Garg et al., 2022).

The *Bcs1l* ^*p.S78G*^ mice are a very severe model of OXPHOS deficiency. It is an interesting question whether MYC upregulation has pathological and perhaps targetable roles in less severe mitochondrial diseases and their experimental models. Proliferating, proliferation-capable, and terminally post-mitotic cells are necessarily very differently affected by MYC upregulation. In post-mitotic cells, such as in skeletal muscle of mitochondrial myopathy patients, MYC upregulation potentially drives adaptive metabolic rewiring and cellular hypertrophy but would not lead to replication issues and DNA damage. One potential consequence of MYC upregulation is a hypermetabolic state. Intriguingly, a recent study identified hypermetabolism and increased energy expenditure as common features of patients with mitochondrial diseases (Sturm et al., 2023). The contribution of potential MYC upregulation to this hypermetabolic state of OXPHOS dysfunction remains, however, speculative at present. MYC is a notorious oncogene, yet mitochondrial disease patients do not show increased cancer risk (Lund et al., 2015). Further studies on the *Bcs1l* ^*p.S78G*^ mice, displaying in some tissues by far the most staggering “cancer-like” MYC upregulation of all mitochondrial disease models, could shed light on the role OXPHOS dysfunction in carcinogenesis if crossed with mice carrying mutant alleles of major tumor suppressor pathways such as p53 or APC (Adenomatosis Polyposis Coli tumor suppressor).

## 7 Concluding words

Here, we argue that MYC could be a functionally important player in mitochondrial diseases, hoping to stimulate researchers of mitochondrial medicine and physiology to study MYC in their models. Given the overarching roles of MYC as a regulator of energy metabolism in cancer and in normal tissue homeostasis, this proposition is not that surprising and has perhaps been hiding in plain sight during the almost 3 decades that mitochondrial diseases have been studied with modern molecular biology tools and methods. What is less clear at this point is the role of MYC in cell proliferation with respect to the widely varying manifestations of mitochondrial diseases in continuously proliferating (e.g., bone marrow) *versus* regeneration-capable (e.g., liver) *versus* permanently postmitotic (e.g., skeletal muscle, brain) tissues that are also metabolically quite different. Our recent findings in the *Bcs1l* ^*p.*S78G^ knock-in mouse model of severe CIII deficiency provide evidence that MYC forces illicit cell cycle entry against depletion of energy and biosynthetic precursors like nucleotides—with catastrophic consequences leading to cellular senescence and progeroid disease. However, the clinical phenotypes of CIII deficiencies caused by other *BCS1L* mutations or by mutations in other genes differ markedly from each other, and there is currently no knowledge about similar mechanisms in these phenotypes. Some key questions that remain to be studied are 1) What signals induce MYC in OXPHOS deficiency, 2) How does MYC contribute to the metabolic shift upon OXPHOS deficiency, and 3) Are the potentially harmful effects of MYC induction a general phenomenon in mitochondrial diseases or do they restrict to those diseases affecting proliferating or regenerating tissues? If MYC-driven DNA damage and cellular senescence occur also as a consequence of other mitochondrial disease mutations than those causing severe CIII deficiency, understanding the role of MYC could enable several novel therapeutic options. While MYC has a reputation of being an undruggable target in cancer, several MYC inhibitors have been developed and some of them have proceeded to clinical trials. Understanding the upstream factors leading to MYC upregulation would further diversify the options to target MYC in mitochondrial disease and potentially also in cancer.
